# Encysted hydrocele of the canal of Nuck in a 7-year-old female: A case report

**DOI:** 10.1016/j.radcr.2025.06.102

**Published:** 2025-07-22

**Authors:** Aqeel Alhashim, Ahmed Almuslim, Ruqaiyah Alhakeem, Hamza Aldossary, Abdullah Alburaih, Fatimah Alsaleh, Tumadhir Alkishi, Ali Alhashim

**Affiliations:** a Department of Radiology, Almoosa Health Group, Al Mubarraz, Kingdom of Saudi Arabia; b Radiology, King Fahad Hospital of the University, Al Khobar, Kingdom of Saudi Arabia; c King Faisal Specialist Hospital and Research Center, Riyadh, Kingdom of Saudi Arabia; d King Faisal University, Hufuf, Kingdom of Saudi Arabia; e Radiology, Royal Commission Hospital in Jubail, Jubail, Kingdom of Saudi Arabia; f Emergency, Alahsa health cluster, Alahsa, Kingdom of Saudi Arabia

**Keywords:** Encysted hydrocele, Hydrocele, Inguinal canal, Groin swelling

## Abstract

A 7-year-old girl presented complaining of right groin swelling for 1 week and underwent routine examination at the emergency department. An ultrasonography reported a well-defined cystic lesion with no internal vascularity, connected to the inguinal canal, and located lateral to the epigastric vessels. Images were not conclusive, and surgical excision was performed 2 days later, with an uneventful postoperative recovery. Intraoperatively, the lesion was identified as an encysted hydrocele of the canal of Nuck. During female development, the parietal peritoneum descended along the round ligament of the uterus through the inguinal ring and into the inguinal canal, which is known as “canal of Nuck.” The round ligament is attached to the uterine cornu adjacent to the origin of the fallopian tube and to the ipsilateral labia majora. If the processus vaginalis does not close and remain patent, this could lead to fluid to pass through and accumulate to form a communicating hydrocele, and the opening is large enough to allow abdominal organs to herniate. This will lead to another differential diagnosis of inguinal swelling, inguinal hernia . This case highlights the importance of considering this rare entity in the differential diagnosis of groin swellings in pediatric females and the role of imaging in guiding management.

## Introduction

Hydrocele of the canal of Nuck is a rare condition in females that presents as an inguino-labial swelling and can be misdiagnosed as an inguinal hernia [1]. This condition results from the incomplete obliteration of the canal of Nuck, a homologue to the processus vaginalis in males [2]. The canal of Nuck is a pouch of parietal peritoneum that accompanies the round ligament as it passes through the inguinal canal [1].

During female development, the parietal peritoneum descends along the round ligament of the uterus, passing through the inguinal ring and into the inguinal canal, forming the canal of Nuck [1,2]. The round ligament extends from the uterine cornu near the fallopian tube origin to the ipsilateral labia majora [1]. Failure of the processus vaginalis to close, also known as a patent processus vaginalis, can lead to fluid accumulation, resulting in a communicating hydrocele [1]. A large opening can also allow abdominal organs to herniate, further complicating the differential diagnosis of inguinal swellings [1].

This case report chronicles the presentation, diagnosis, and surgical management of a 7-year-old female with an encysted hydrocele of the canal of Nuck, highlighting the importance of considering this entity in the differential diagnosis of groin masses in young females.

## Case report

A 7-year-old female patient presented to Almoosa Specialist Hospital in Al-Ahsa with a progressively enlarging, painless lump in the right groin that had been present for the past week. Upon initial assessment, the patient was vitally stable and afebrile, showing no signs of systemic distress. A thorough physical examination revealed an oval-shaped, well-circumscribed, nontender, and fluctuant swelling located in the right inguinal region. Notably, there were no skin changes, erythema, or inflammation around the swelling. The patient did not report any symptoms of vomiting or urinary issues. Additionally, the parents did not report any history of trauma or prior surgical procedures. The swelling was nonreducible, and there was no palpable cough impulse, making the likelihood of an inguinal hernia less probable.

**Fig. 1:**
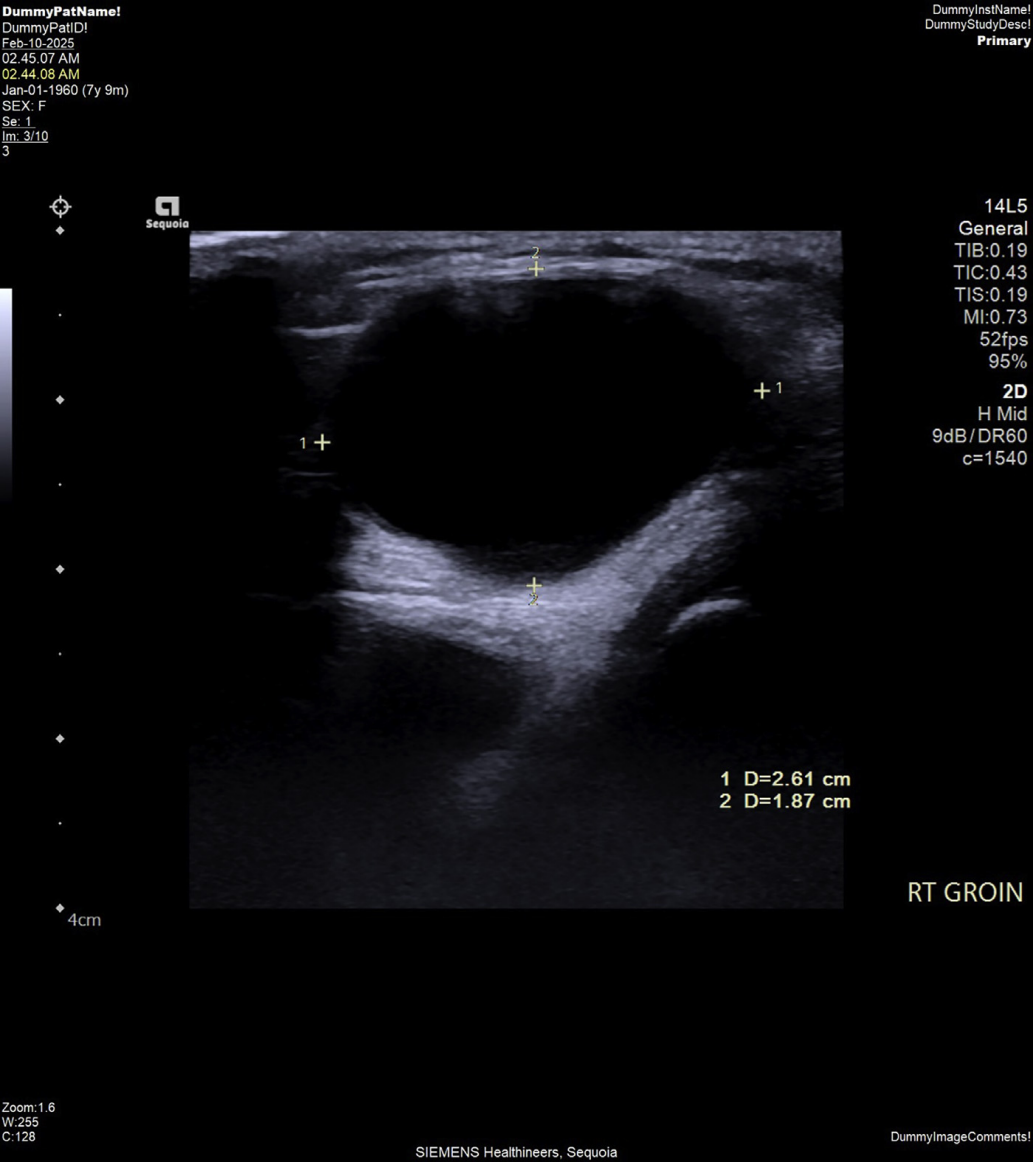
Gray-scale ultrasound image of the right groin showing an elliptical anechoic cystic lesion positioned laterally to the epigastric vessels measuring 2.6 × 1.9 cm. No herniation of pelvic organs or bowel loops was seen.

**Fig. 2:**
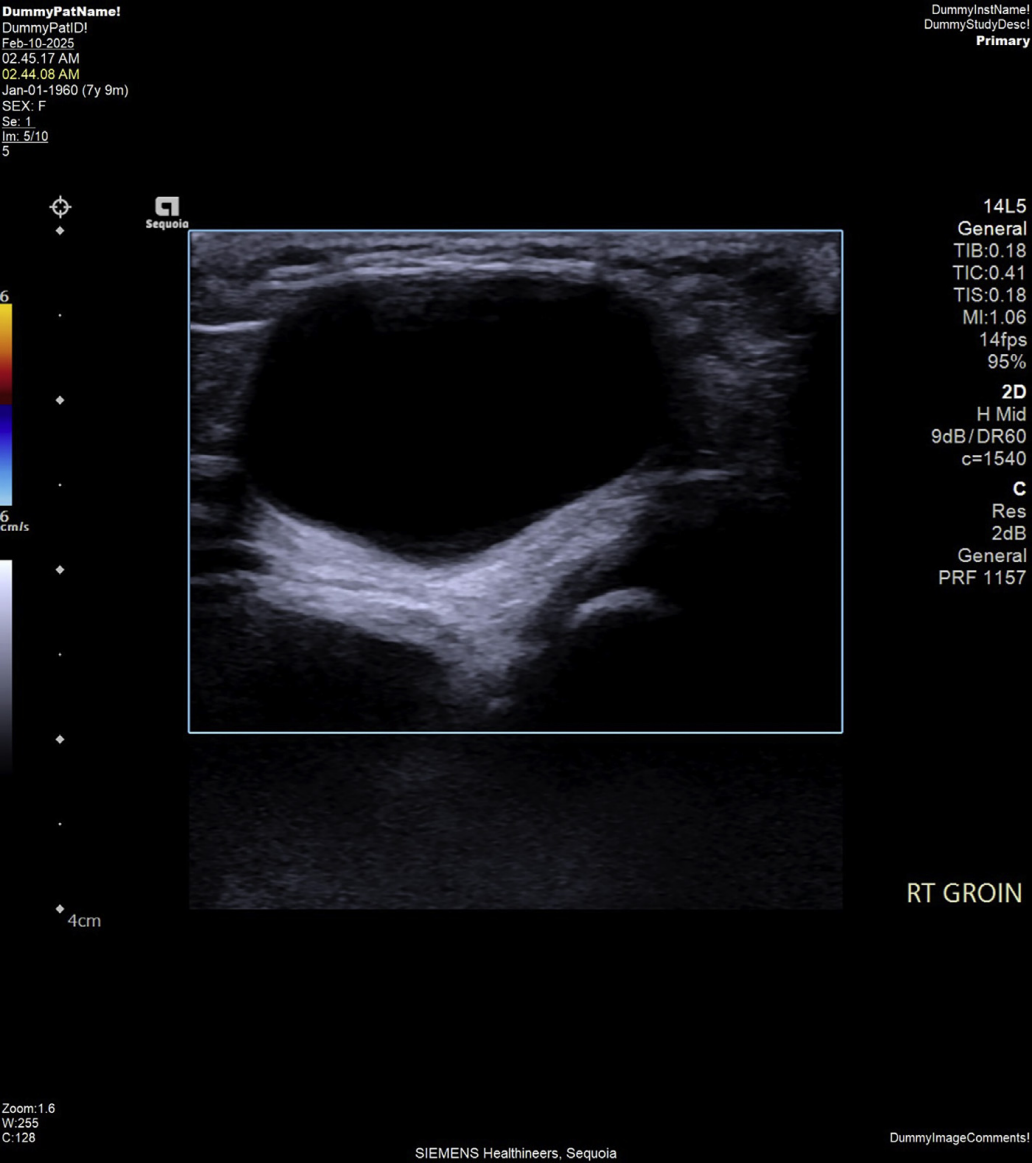
Color Doppler ultrasound image of the right groin showing a fluid-filled cystic lesion with no notable vascular uptake.

To further investigate, an ultrasonography of the right inguinal region was performed, which revealed a 2.6 × 1.9 cm fan anechoic cystic lesion situated laterally to the epigastric vessels (Fig. 1). Doppler imaging showed no vascularity within the lesion (Fig. 2). These sonographic findings were consistent with a hydrocele of the canal of Nuck. Based on these results, pediatric surgeon on duty was consulted for further evaluation. After a detailed consultation, the decision was made to proceed with surgical excision, and the patient was scheduled for the procedure 2 days later.

The surgical excision was carried out under general anesthesia. A standard inguinal incision was made, and the cystic lesion was carefully identified. The lesion was then meticulously dissected and excised in its entirety to prevent any risk of recurrence. The processus vaginalis was ligated at the level of the deep inguinal ring. The procedure was completed smoothly, with no intraoperative complications.

Following surgery, the patient experienced an uneventful recovery. There were no immediate or delayed postoperative complications. A follow-up appointment 2 weeks postoperatively showed a well-healed surgical site, with no recurrence of the swelling. Histopathological examination of the excised lesion confirmed the diagnosis of an encysted hydrocele of the canal of Nuck, thereby confirming the accuracy of the preoperative diagnosis.

## Discussion

Hydrocele of the Canal of Nuck was first reported by Dutch anatomist Anton Nuck in 1691 [1]. Hydrocele of the Canal of Nuck is a rare condition in female children, results from the failure of the processus vaginalis to obliterate, leading to fluid accumulation within the canal [3]. This condition is classified into 3 types as follows: the most common type is the first type, which is partial obliteration of just the proximal portion of the canal of Nuck, forming an encysted hydrocele where there is no communication between the hydrocele and the peritoneal cavity. The second type is a communicating hydrocele where the canal of Nuck is completely patent, which looks like the hydrocele seen in the male population. The third type is a combination of the first 2 types; the proximal part of the sac is retroperitoneal, and the distal part is in the inguinal canal; the hydrocele is compressed by the deep inguinal ring, which would appear as an hourglass making it difficult to distinguish it from inguinal hernia [3,4]. Hydrocele of the canal of Nuck consider a rare condition according to a study done in July 1998-March 2013 (0.76% rate in a large series of 787 inguinal surgical explorations) [5] giving its rarity it lead to commonly misdiagnosis with other differential diagnosis for groin swellings in females may including indirect or femoral inguinal hernias, enlarged lymph nodes (buboes), Bartholin’s gland cysts, hematomas following trauma, lipomas, vascular aneurysms, and less commonly, entities like cystic lymphangiomas, metastatic neuroblastoma, ganglion cysts, leiomyomas, soft tissue sarcomas, round ligament endometriosis, or epidermoid cysts [1].

Clinically, the hydrocele of the canal of Nuck presents as painless or uncomfortable, nonpulsatile mobile inguinal swelling with no evidence of nausea or vomiting [4]. Making a definitive diagnosis of a hydrocele of the canal of Nuck clinically is quite challenging which would lead to misdiagnosis and wrong management, therefore, utilizing diagnostic imaging is a must for investigations starting with ultrasound which is considered the optimum modality for early diagnosis, which would demonstrate a well-defined unilocular or multilocular, anechoic or hypoechoic lesion with posterior acoustic enhancement as in our case which presented with typical features of well-defined, anechoic cystic lesion lacking internal vascularity [6]. High-resolution sonographic imaging could be enough to establish the final diagnosis. However, if the ultrasound finding is inconclusive, Magnetic resonance imaging could help reach the diagnosis, in which the hydrocele typically demonstrates low signal intensity in T1-weighted sequences and high signal intensity in T2-weighted images [6,7].

For those with low-risk lesions and asymptomatic patients, observation and conservative management is recommended, however, complications related to hydrocele of the canal of Nuck are possible, such as developing infected hydrocele, hemorrhage, or abscess formation. Therefore, for symptomatic patients or in complicated cysts, surgical management is the optimal treatment option. There is no difference in the effectiveness of laparoscopic or open surgical procedures. [5].

## Conclusion

Despite the rarity of hydrocele of the canal of Nuck, it is a must to consider it in the differential diagnosis of any female pediatric patient presenting with inguinolabial swelling or masses. Despite its potential for significant morbidity if left untreated, it is often under-recognized and misdiagnosed. Physical examination alone often is not sufficient for diagnosis; utilizing high-resolution ultrasound imaging or MRI is essential to confirm the diagnosis preoperatively. The treatment of choice is complete surgical excision, which involves carefully dissecting the hydrocele from the round ligament and ligating the canal of Nuck near the deep inguinal ring [7].

## Patient consent

Written consent was obtained from the patient’s guardian for the publication of this article.
